# Foot-and-Mouth Disease Virus Serotype A in Egypt

**DOI:** 10.3201/eid1310.070252

**Published:** 2007-10

**Authors:** Nick J. Knowles, Jemma Wadsworth, Scott M. Reid, Katherine G. Swabey, Alaa A. El-Kholy, Adel Omar Abd El-Rahman, Hatem M. Soliman, Katja Ebert, Nigel P. Ferris, Geoffrey H. Hutchings, Robert J. Statham, Donald P. King, David J. Paton

**Affiliations:** * Institute for Animal Health, Surrey, United Kingdom; † Veterinary Sera and Vaccines Research Institute, Cairo, Egypt

**Keywords:** Aphthovirus, epidemiology, molecular, foot-and-mouth disease virus, molecular sequence data, phylogeny, picornaviridae, dispatch

## Abstract

We describe the characterization of a foot-and-mouth disease (FMD) serotype A virus responsible for recent outbreaks of disease in Egypt. Phylogenetic analysis of VP1 nucleotide sequences demonstrated a close relationship to recent FMD virus isolates from East Africa, rather than to viruses currently circulating in the Middle East.

Foot-and-mouth disease (FMD) is caused by 7 immunologically distinct serotypes, O, A, C, Asia 1, South African Territories (SAT) 1, SAT 2, and SAT 3, which belong to the species *Foot-and-mouth disease virus* (genus *Aphthovirus*, family *Picornaviridae*). Several of these serotypes circulate currently or periodically in the Middle East and North Africa (*1*). In Egypt, routine prophylactic vaccination has been conducted with a locally produced serotype O vaccine. The last outbreak of serotype O was in June 2000, and other serotypes have not been reported since 1972 when serotype A occurred (*2*). This report describes an FMD serotype A virus responsible for recent outbreaks of disease in Egypt.

Clinical cases of FMD were first recognized on January 22, 2006, on a cattle farm at El Etehad in Ismailia, northeastern Egypt (Figure 1). Samples were submitted for laboratory investigation and serotype determination by using virus isolation, antigen ELISA, and reverse transcription–PCR (RT-PCR). Initial testing with antigen ELISA and RT-PCR assays suggested that multiple FMD virus (FMDV) serotypes may have been involved in the outbreak (data not shown), although only type A was later confirmed. On February 15, 2006, the Agriculture Ministry in Egypt notified international public health authorities (by reporting to the World Organization for Animal Health [OIE]) of 6 outbreaks of FMDV caused by serotype A in Ismailia and 12 additional outbreaks in 7 other Egyptian governorates: Alexandria (2 outbreaks), Behera (1 outbreak), Cairo (1 outbreak), Dakahlia (1 outbreak), Dumyat (5 outbreaks), Fayum (1 outbreak), and Menofia (1 outbreak). By April 6, 2006, 34 outbreaks of disease had been reported that affected >7,500 animals and involved an additional governorate (Kalubia). Most (96.7%) clinical FMD cases involved cattle; 411 cattle (mainly calves) reportedly died. Attempts to control the outbreaks were hampered by lack of an appropriate vaccine and concurrent outbreaks of highly pathogenic avian influenza. FMD became widespread in Egypt, with the following numbers of animals affected per month: 6,189 (January), 1,858 (February), 3,035 (March), 401 (April), and 297 (May). A locally produced bivalent FMDV vaccine, containing both O_1_ and A/Egypt/2006 isolates, was released in mid-May 2006 for the first time in Egypt. No new cases have been reported since July 2006.

**Figure 1:**
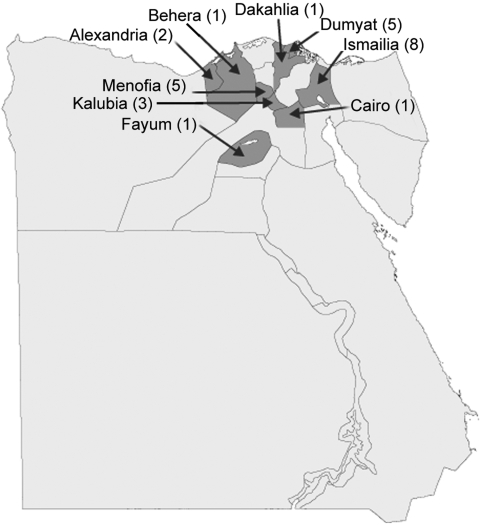
Locations and number of cases in the initial outbreaks of foot-and-mouth disease, Egypt, 2006**.**

## The Study

Clinical material from 5 cases (collected from 3 separate locations in Egypt; Table 1) was sent to the Food and Agricultural Organization of the United Nations (FAO) World Reference Laboratory for FMD (WRLFMD) at the Institute for Animal Health, Pirbright, United Kingdom, for confirmatory diagnosis and characterization of the causative FMDV strain(s). The possibility that these samples contained multiple FMDV serotypes was also investigated. FMDV isolates causing cytopathic effects in primary bovine thyroid (BTy) cell cultures were generated from all samples. The cell culture–grown virus isolates and original clinical submissions were identified as FMDV serotype A by antigen-detection ELISA (*3*).

**Table 1:** Foot-and-mouth disease type A viruses examined in the study

WRLFMD ref. no. or virus name*	Location	Date collected	Species	GenBank accession no.
A/ARG/2000	Argentina	2000	Not known	AY593782
A/Trenquelauquen/ ARG/2001	Trenquelauquen, Argentina	Mar 31, 2001	Bovine	AY593786
A_24_/Cruzeiro/BRA/55	Cruzeiro, Brazil	1955	Bovine	AJ251476
A/CAR/15/2000	Lahore Vina, Vina, Adamawa, Cameroon	2000	Bovine	EF208755
A/EGY/1/72	Alexandria, Egypt	May 13, 1972	Bovine	EF208756
A/EGY/1/2006	Ismailia, Egypt	Feb 9, 2006	Bovine	EF208757
A/EGY/2/2006	Ismailia, Egypt	Feb 9, 2006	Bovine	EF208758
A/EGY/3/2006	Ismailia, Egypt	Feb 9, 2006	Bovine	EF208759
A/EGY/4/2006	Fayoum, Egypt	Feb 16, 2006	Bovine	EF208760
A/EGY/5/2006	Domiat, Egypt	Feb 19, 2006	Bovine	EF208761
A/ETH/7/92	Shena, Ethiopia	Oct 3, 1992	Bovine	EF208765
A/ETH/1/94	Highland areas of Eastern Ethiopia	Feb 2, 1994	Bovine	EF208766
A/ETH/23/94	Nazret, East Shoa, Ethiopia	Mar 9, 1994	Not known	EF208767
A_5_/Allier/FRA/60	Allier, France	1960	Bovine	AY593780
A/GAM/44/98	Gambia	Feb 4, 1998	Not known	EF208768
A_10_/HOL/42	Groot-Ammers, the Netherlands	1942	Bovine	M20715
A/IND/17/77†	Tamil Nadu, India	1977	Bovine	AF204108
A/IRN/2/87	Mardabad, Kardaj, Tehran, Iran	Mar 11,1987	Bovine	EF208770
A/IRN/1/96	Zarnan, Shahriar, Tehran, Iran	Nov 13, 1996	Bovine	EF208771
A/IRN/22/99	Tabriz, East Azerbaijan Province, Iran	1999	Bovine	EF208772
A/IRN/1/2005	Ghalch-Sadri, Qom, Qom Province, Iran	Apr 4, 2005	Bovine	EF208769
A_22_/IRQ/24/64	Mosul, Iraq	1964	Bovine	AJ251474
A_21_/Lumbwa/KEN/64	Lumbwa, Kenya	1964	Bovine	AY593761
A_23_/Kitale/KEN/64	Kitale, Kenya	1964	Bovine	AY593766
A/KEN/15/98	Meru, Kenya	Sep 8, 1998	Bovine	EF208774
A/KEN/16/98A	Nakuru, Kenya	Sep 15, 1998	Bovine	EF208775
A/KEN/29/2005	Embu, Eastern Province, Kenya	Aug 24, 2005	Bovine	EF208773
A/MAI/2/97	Mali	Not known	Not known	EF208776
A_15_/Bangkok/TAI/60	Bangkok, Thailand	1960	Bovine	AY593755
A/TAI/118/87†	Sara Buri, Thailand	1987	Not known	EF208777
A/TAI/2/97	Thailand	1997	Not known	EF208778
A_12_/UK/119/32	Kent, United Kingdom	1932	Bovine	AY593752

*WRLFMD, World Reference Laboratory for Foot-and-Mouth Disease. †Not a WRLFMD reference no.

**Figure 2:**
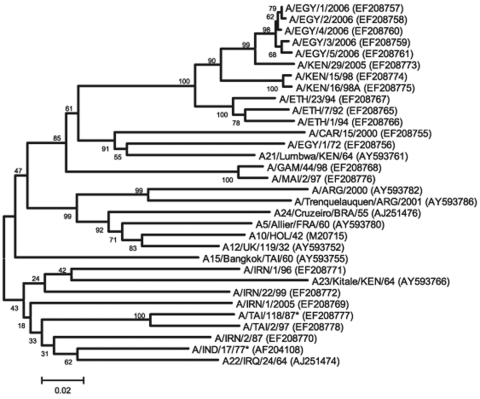
Midpoint-rooted neighbor-joining tree showing the relationships between the A Egypt 2006 virus isolates and other contemporary and reference viruses. Numbers indicate the percentage occurrence of the branches by the bootstrap resampling method. *Reference number not assigned by the World Reference Laboratory for Foot-and-Mouth Disease**.**

Total RNA was extracted from the first virus passage on BTy cells by using RNeasy kits (QIAGEN, Crawley, UK) for all 5 samples (EGY/1/2006–EGY/5/2006) (*4*). The complete VP1 region of the genome was amplified by RT-PCR by using 2 primer sets (A-1C562F/EUR-2B52R and A-1C612F/EUR-2B52R; Table 2) and the following thermal profile: 42°C for 30 min; 94°C for 5 min; 35 cycles of 94°C for 60 s, 55°C for 60 s, and 72°C for 90 s, followed by a final extension of 72°C for 5 min. The sequence of each amplicon was determined by cycle sequencing as previously described (*4*) but with the primers NK72, A-1C612F, and A-1D523R (Table 2). An unrooted neighbor-joining tree was constructed by using MEGA version 3.1 (*5*). The robustness of the tree topology was assessed with 1,000 bootstrap replicates as implemented in the program. Additionally, maximum parsimony (MEGA 3.1), minimum evolution (MEGA 3.1), and maximum likelihood (TREE-PUZZLE 5.2; [*6*]) trees were constructed; all 4 methods gave similar tree topologies (data not shown). Egyptian sequences shared a closer phylogenetic relationship with recent and historical isolates from East Africa rather than with contemporary serotype A viruses emerging from Iran, currently circulating in the Middle East and European Turkey (Figure 2).

**Table 2:** Oligonucleotide primers used for RT-PCR and sequencing*

Primer name	Primer sequence (5′→3′)	Sense	Gene	Position†
A-1C562F	TACCAAATTACACACGGGAA	Forward	VP3	3123–3142
A-1C612F	TAGCGCCGGCAAAGACTTTGA	Forward	VP3	3173–3193
EUR-2B52R	GACATGTCCTCCTGCATCTGGTTGAT	Reverse	2B	3963–3988
NK72	GAAGGGCCCAGGGTTGGACTC	Reverse	2A/2B	3897–3917
A-1D523R	CGTTTCATRCGCACRAGRA	Reverse	VP1	3748–3766

*RT-PCR, reverse transcription–PCR. †Position on the genome of A_21_/Lumbwa/KEN/64 (GenBank accession no. AY593761).

Other conventional “typing” PCRs were performed to investigate whether additional FMDV serotypes were present in these samples. A multiplex agarose gel–based RT-PCR that targeted VP1 of O, A, C, and Asia 1 (primers P33, P38, P87–92, P40, P74–77) (*7*) generated a single band corresponding to the size expected (702 bp) for serotype A for all 5 samples (data not shown). In addition, a cocktail of primers (P1, P126, P150–153, P130, P159–161, P168–170) (*7*) recognizing VP1 of SAT1–3 serotypes did not show any bands after RT-PCR with these samples. However, amplicons of correct size (715 bp) were obtained after RT-PCR with samples EGY/1/2006, EGY/3/2006, and EGY/5/2006 when an additional primer set for SAT 1–3 VP1 (1D209F/2B208R) was used (*8*). Subsequent analysis of these SAT amplicons generated sequences that corresponded to serotype A, identical to the complete VP1 sequences of A/EGY/1/2006 and A/EGY/2/2006. Together, these findings support the conclusion that FMDV corresponding to a single serotype A was present in this material.

For vaccine selection, serologic tests were conducted to evaluate the extent of in vitro cross-neutralization of A/EGY/1/2006 and A/EGY/2/2006 by antisera produced against available FMDV vaccine strains (*9*). The match (r_1_ value) against the vaccine strains A_22_/Iraq/64 and A/Iran/96 that are regularly used elsewhere in the Middle East was less than the cut-off value of 0.3 (r_1_ = 0.23 and 0.24, respectively), whereas an acceptable match (r_1_ = 0.42) was found against the A/Eritrea/98 vaccine strain that is of East African origin. However, A/Eritrea/98 vaccine is not in routine production nor held in vaccine reserves and was therefore not available for immediate supply. A recent in vivo study demonstrated that a high potency A_22_/Iraq/64 vaccine could provide clinical protection against challenge with the new A/EGY/2006 virus (B. Haas, pers. comm., 2006). High-potency vaccines are known to protect even when relationship values are lower than the normal cut-off values (*10*).

## Conclusions

Local interpretation of agarose-based RT-PCR assays and sequence data led the Egyptian authorities to initially suspect the involvement of at least 2 serotypes, A and SAT 2. However, tests performed at the WRLFMD conclusively showed the presence of a single serotype, A, in the samples received from Egypt. Unofficial reports suggest that the disease was introduced by animals imported from Ethiopia for slaughter (*11*). This hypothesis is consistent with the results of the molecular typing, which suggested a relation between strains of Egyptian and East African origin. The molecular typing confirms only that through the trade in live cattle, an East African type A strain was introduced, which was not contained at the quarantine station. The origin of the infection is unclear, since the animals in quarantine may have acquired infection at various points during shipment, including possible contaminated pens or other animals on board the ship, at the port before loading, or in transit from Ethiopia to the port of loading. Veterinary inspection of the quarantined animals also detected cases of lumpy skin disease (LSD), and possibly the origin of the LSD epidemic in Egypt in 2006 may relate to the Ethiopian animal trade, which is supported by the reports of LSD epidemics in Ethiopia in 2005. Undoubtedly, the lack of reporting of disease preimportation or at the quarantine stations did not assist the authorities in controlling the disease. Because imported animals may acquire infection at any point up until their arrival, they must be vaccinated and tested for the absence of FMDV nonstructural proteins.
